# Fluorescence Spectrometric Determination of Nonfluorescent Compounds via Molecular Fragmentation

**DOI:** 10.6028/jres.093.108

**Published:** 1988-06-01

**Authors:** E. L. Wehry

**Affiliations:** Department of Chemistry, University of Tennessee Knoxville, TN 37996

Molecular fluorescence spectrometry offers several important analytical advantages, including (a) achievement of very low limits of detection for intensely fluorescent analytes, (b) applicability to determine individual constituents of multicomponent samples without prior separation, (c) applicability to remote detection of analytes via fiber optic and/or laser probes, and (d) the high information content of luminescence measurements (excitation and emission spectra, decay times, polarization).

A principal shortcoming of fluorescence spectrometry is the fact that most organic and inorganic molecules exhibit low luminescence quantum yields and thus cannot be detected by fluorometry without first being converted to fluorescent derivatives. Accordingly, many procedures for derivatizing nonfluorescent analytes to form luminescent products have been devised.

Our work takes a somewhat different approach to fluorometric detection of nonfluorescent molecules. We exploit the fact that many small molecular fragments are intensely luminescent, including such common species as NH, OH, CN, CH, PO, and SH, as well as many atoms. Fluorescent fragments can be produced from virtually any nonfluorescent molecule. For analytical purposes, the key is to perform the fragmentation in a sufficiently reproducible manner that the fluorescence intensity of a fragment species can be related to the concentration of parent molecule present in the initial sample.

We presently perform molecular fragmentations in the gas phase, to avoid “cage effects” which often decrease the efficiencies of fragmentation processes in liquid or solid media to unacceptably low levels. From among the large number of possible techniques for molecular fragmentation, we have chosen laser photolysis (LP) and electron impact (EI) as the most promising methods.

In LP experiments, gaseous samples are fragmented by either the beam from an excimer laser (193 nm) or tunable radiation from an excimer-pumped dye laser. In EI methods, molecules are fragmented by 100-eV electrons from a conventional heated-filament electron gun. A certain fraction of fragment species are produced in electronically excited states and emit directly. If needed, a “probe” laser can be used to excite luminescence from fragments formed in their ground electronic states by LP or EI. Vacuum systems comparable in design and performance to those found in conventional mass spectrometers are usually used, though LP fragmentations can also in principle be applied directly to remote atmospheric sensing. Conventional fluorescence measurement and signal-processing techniques are used.

We will describe studies of the analytical characteristics (limits of detection, linear dynamic range, precision) for LP and EI fragmentation-fluorometric detection of a variety of nonfluorescent organic and organometallic compounds. The possibility of using low-temperature techniques (matrix isolation or supersonic expansion) to increase the selectivity of such measurements will be described. The complementarity of these measurements to mass spectrometry will be emphasized (most of the intensely emissive fragment species are neutrals, whereas ionic fragments are detected in mass spectrometry).

